# Supplementary material for: The Impact of COVID-19 on Multidrug-Resistant Bacteria at a Slovenian Tertiary Medical Center
Source: Antibiotics (Basel). 2024 Feb 23;13(3):214. doi: 10.3390/antibiotics13030214 (PMC10967484; doi:10.3390/antibiotics13030214)
Supplement: Supplementary file 1 [file antibiotics-13-00214-s001.zip › antibiotics-2828929-supplementary.pdf]

Microbiological data: The data on bacterial isolates and their antimicrobial susceptibility were retrieved from the laboratory information system (SRC Infonet, Slovenia) at the IMI.

The isolates were identified using mass spectrometry MALDI-TOF LT Microflex (Bruker Daltonics, Bremen). Data were collected on the following bacteria: *A. baumannii*, *E. faecium*, *E. coli*, *K. pneumoniae*, *P. aeruginosa*, and *S. aureus*. Antimicrobial susceptibility was determined using disk diffusion according to EUCAST guidelines with the exception of glycopeptide susceptibility for *S. aureus* where minimal inhibitory concentration was determined using gradient diffusion tests [25]. Resistance phenotypes were determined using EUCAST guidelines [26]. The following multidrug-resistant bacteria (MDRB) were included in the study: carbapenem-resistant *A. baumannii* (CRAb), vancomycin-resistant *E. faecium* (VRE-EFA), extended-spectrum beta-lactamase–producing *E. coli* (ESBL-EC) and *K. pneumoniae* (ESBL-KPN), carbapenem-resistant *K. pneumoniae* (CRE-KPN), beta-lactam resistant *P. aeruginosa* (CRPs-PA), methicillin-resistant and *S. aureus* (MRSA). CRPs-PA was defined according to Slovenian national guidelines as an isolate resistant to all classes of antipseudomonal beta-lactams [10].

Table S1. Bacteria included in the study with antibiotic panels, resistance phenotypes indicator antibiotic and corresponding multidrug-resistant bacteria (MDRB).

Bacteria	Antibiotic panels	Resistance phenotypes - indicator antibiotic	MDRB	MDRB abbrevation
Acinetobacter baumannii	imipenem, gentamycin, amikacin, ciprofloxacin, levofloxacin, trimetoprim-sulphametoxasole	imipenem	carbapenem-resistant *A. baumannii*	CRAb
Enterococcus faecium	Ampicillin, gentamycin (test for high-level aminoglycoside resistance), vancomycin, teicoplanin	vancomycin	vancomycin-resistant *E. faecium*	VRE-EFA
Escherichia coli	Ampicillin, amoxicillin-clavulanic acid, piperacillin-tazobactam, cefuroxime, cefotaxime, ceftazidime, cefepime, ertapenem, imipenem, meropenem, gentamycin, amikacin, ciprofloxacin, levofloxacin, trimetoprim-sulphametoxasole	cefotaxime, ceftazidime	extended-spectrum beta-lactamase–producing *E. coli*	ESBL-EC
Klebsiella pneumoniae	Ampicillin, amoxicillin-clavulanic acid, piperacillin-tazobactam, cefuroxime, cefotaxime, ceftazidime, cefepime, ertapenem, imipenem, meropenem, gentamycin, amikacin, ciprofloxacin, levofloxacin, trimetoprim-sulphametoxasole	cefotaxime, ceftazidime	extended-spectrum beta-lactamase–producing *K. pneumoniae*	ESBL-KPN
Klebsiella pneumoniae		meropenem	carbapenem-resistant *K. pneumoniae*	CRE-KPN
Pseudomonas aeruginosa	piperacillin-tazobactam, ceftazidime, cefepime, imipenem, meropenem, amikacin, ciprofloxacin, levofloxacin	piperacillin-tazobactam, ceftazidime, cefepime, imipenem, meropenem*	beta-lactam resistant *P. aeruginosa**	CRPs-PA
Staphylococcus aureus	benzylpenicillin, cefoxitin, clindamycin, erythromycin, rifampicin, ciprofloxacin, tetracycline, trimetoprim-sulphametoxasole, gentamycin, linezolid; vancomycin and teicoplanin	cefoxitin	methicillin-resistant *S. aureus*	MRSA

* CRPs-PA defined according to Slovenian national guidelines as an isolate resistant to all classes of antipseudomonal beta-lactams [10].
